# Supplementary material for: The art of using t-SNE for single-cell transcriptomics
Source: Nat Commun. 2019 Nov 28;10:5416. doi: 10.1038/s41467-019-13056-x (PMC6882829; doi:10.1038/s41467-019-13056-x)
Supplement: Supplementary file 1 — Supplementary Information [file 41467_2019_13056_MOESM1_ESM.pdf]

The art of using t-SNE for single-cell transcriptomics. Supplementary Information

Dmitry Kobak, Philipp Berens

August 2019

Supplementary Figures

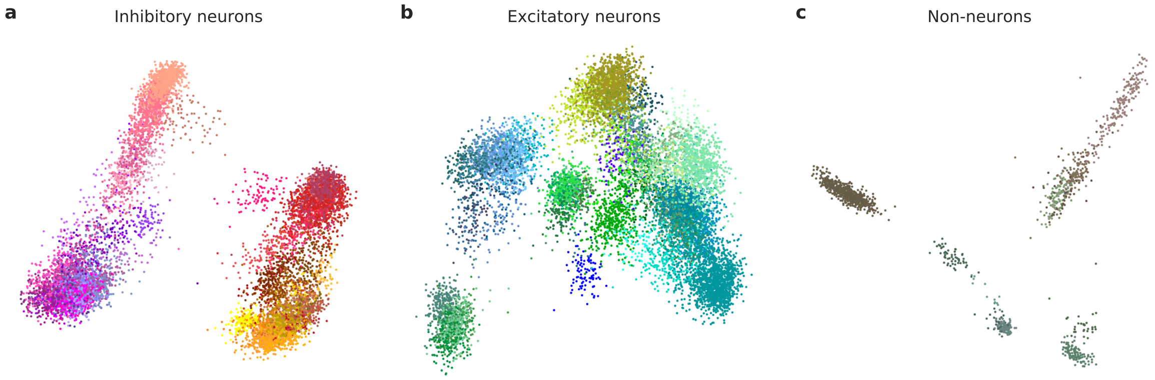

Supplementary Figure 1: Major cell classes in the Tasic et al. 2018 data set. Cluster colours as in Figure 2. In each case PCA makes some of the within-class internal structure obvious. **(a)** PCA of the inhibitory neurons. The scatter plot shows two main principal components. Purple: *Vip* neurons. Salmon: *Lamp5* neurons. Red: *Pvalb* neurons. Orange: *Sst* neurons. **(b)** PCA of the excitatory neurons. **(c)** PCA of the non-neural cells.

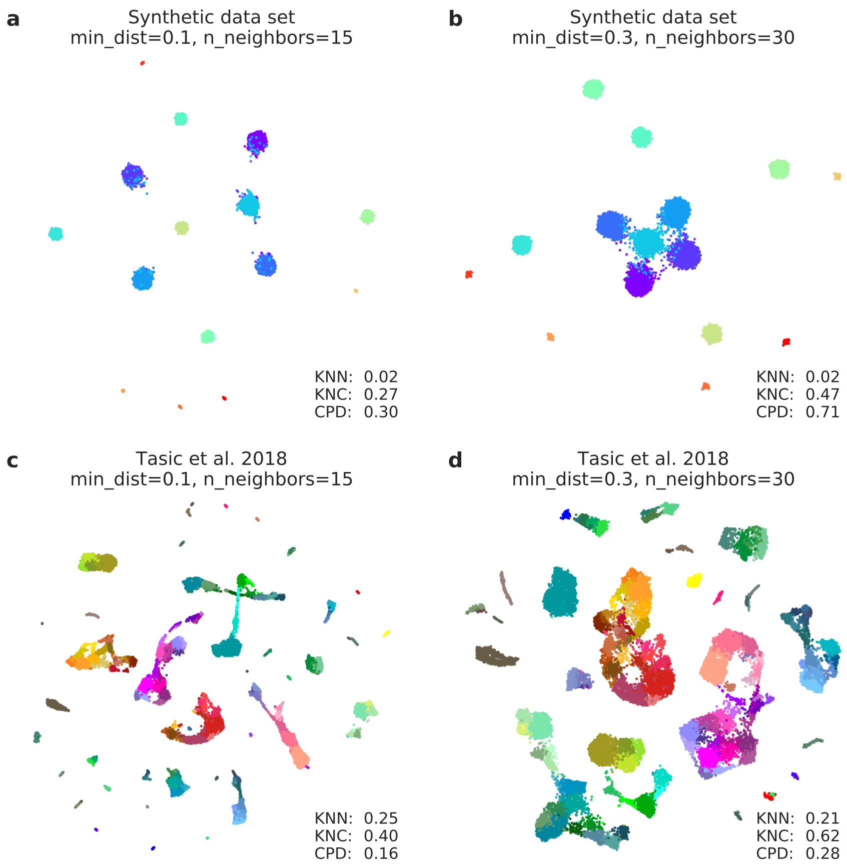

Supplementary Figure 2: UMAP embeddings of the small data sets. Cluster colours as in Figure 1 and Figure 2. For all embeddings, random seed was set to 1. **(a)** UMAP embedding of the synthetic data set with the default parameters (`min_dist=0.1, n_neighbors=15`). **(b)** UMAP embedding of the synthetic data set with parameters `min_dist=0.5, n_neighbors=30` that tend to produce less fragmented embeddings. In both (a) and (b) all three metrics are worse than in our t-SNE embedding in Figure 1f: KNN=0.11, KNC=0.82, CPD=0.74. **(c)** UMAP embedding of the Tasic et al. 2018 data set with the default parameters (`min_dist=0.1, n_neighbors=15`). **(d)** UMAP embedding of the Tasic et al. 2018 data set with parameters `min_dist=0.5, n_neighbors=30`. In both (c) and (d) all three metrics are worse than in our t-SNE embedding in Figure 2f: KNN=0.41, KNC=0.68, CPD=0.53.

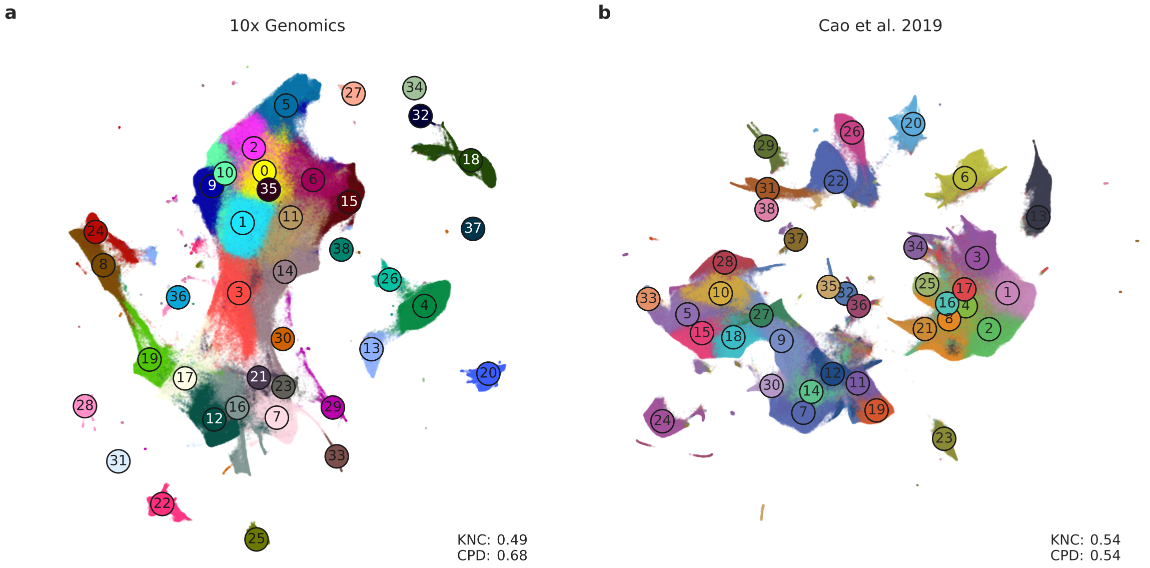

Supplementary Figure 3: UMAP embeddings of the large data sets. Cluster colours as in Figure 7 and Figure 9. For all embeddings, random seed was set to 1. **(a)** UMAP embedding of the 10x Genomics data set, default parameters. The interneuron clusters (##8, 24, 19, 26, 4, 13) are scattered across the embedding. **(b)** UMAP embedding of the Cao et al. data set, default parameters. Note that cluster #13 (myocytes) appears isolated, even though it belongs to the mesenchymal trajectory.

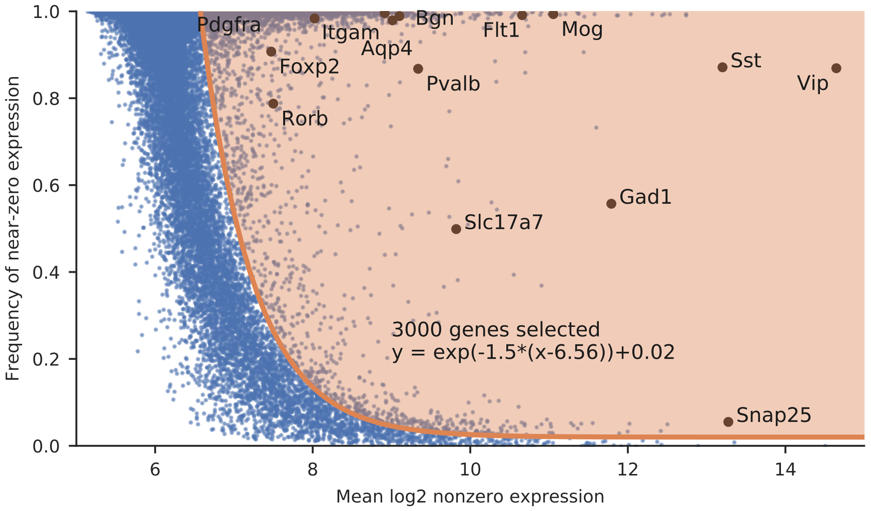

Supplementary Figure 4: Feature selection. Our feature selection procedure illustrated for the Tasic et al. (2018) data set. Black dots show well-known marker genes, taken from Figure 1c of Tasic et al. (2016). Any good feature selection procedure should confidently select all of them.
